# Investigation of the differences in characteristics of Welsh farming households with and without off-farm income

**DOI:** 10.23889/ijpds.v9i5.2827

**Published:** 2024-09-18

**Authors:** Jenny Thyer, Sian Morrison-Rees, Sean Scully, Paul Caskie

**Affiliations:** 1 Swansea University; 2 Agri-Food and BioSciences Institute

## Objective

To examine the characteristics of farming households that have no off-farm income, and how they compare to farming households containing at least one person employed in a non-farming occupation.

## Approach

This research uses the AD|ARC dataset for Wales held in the SAIL databank at Swansea University. This Research Ready Dataset, created by the research team, is comprised of population census data and existing agricultural datasets.

Individuals were split into two groups, those in households where all individuals were primarily employed in farming or unemployed, and those in households where at least one individual was in a non-farming occupation.

## Results

Comparing these groups, our findings show:

- There are 8135 farming households with at least one person in a non-farming occupation, and 5415 households with no off-farm income.
- Households with off-farm income are on average 65% larger than households with no off-farm income.
- 33% of farming only households consist of a single person; 73% of these farmers are male, and the average age is 62.
- The average age for farming-only households is 45, and the average age of households with non-farming occupations is 36.
- We will present detailed findings on the socio-economic and farm business characteristics of these groups.

## Conclusion

Understanding differences between households with and without off-farm income provides insights on farm types and households most impacted by changes in agricultural policies and subsidies. This can inform policy design and help authorities deliver commitments on just transitions in a targeted and efficient manner.

